# Pareto-Optimized Non-Negative Matrix Factorization Approach to the Cleaning of Alaryngeal Speech Signals

**DOI:** 10.3390/cancers15143644

**Published:** 2023-07-16

**Authors:** Rytis Maskeliūnas, Robertas Damaševičius, Audrius Kulikajevas, Kipras Pribuišis, Nora Ulozaitė-Stanienė, Virgilijus Uloza

**Affiliations:** 1 Faculty of Informatics, Kaunas University of Technology, 44249 Kaunas, Lithuania; robertas.damasevicius@ktu.lt (R.D.); audrius.kulikajevas@ktu.lt (A.K.); 2 Department of Otorhinolaryngology, Academy of Medicine, Lithuanian University of Health Sciences, 44240 Kaunas, Lithuania; kipras.pribuisis@lsmuni.lt (K.P.); nora.ulozaite@lsmuni.lt (N.U.-S.); virgilijus.ulozas@lsmuni.lt (V.U.)

**Keywords:** alaryngeal, voice quality, voice cleaning, voice disorders, Pareto optimization

## Abstract

**Simple Summary:**

This paper introduces a new method for cleaning impaired speech by combining Pareto-optimized deep learning with Non-negative Matrix Factorization (NMF). The approach effectively reduces noise in impaired speech while preserving the desired speech quality. The method involves calculating the spectrogram of a noisy voice clip, determining a noise threshold, computing a noise-to-signal mask, and smoothing it to avoid abrupt transitions. Using a Pareto-optimized NMF, the modified spectrogram is decomposed into basis functions and weights, allowing for reconstruction of the clean speech spectrogram. The final result is a noise-reduced waveform achieved by inverting the clean speech spectrogram. Experimental results validate the method’s effectiveness in cleaning alaryngeal speech signals, indicating its potential for real-world applications.

**Abstract:**

The problem of cleaning impaired speech is crucial for various applications such as speech recognition, telecommunication, and assistive technologies. In this paper, we propose a novel approach that combines Pareto-optimized deep learning with non-negative matrix factorization (NMF) to effectively reduce noise in impaired speech signals while preserving the quality of the desired speech. Our method begins by calculating the spectrogram of a noisy voice clip and extracting frequency statistics. A threshold is then determined based on the desired noise sensitivity, and a noise-to-signal mask is computed. This mask is smoothed to avoid abrupt transitions in noise levels, and the modified spectrogram is obtained by applying the smoothed mask to the signal spectrogram. We then employ a Pareto-optimized NMF to decompose the modified spectrogram into basis functions and corresponding weights, which are used to reconstruct the clean speech spectrogram. The final noise-reduced waveform is obtained by inverting the clean speech spectrogram. Our proposed method achieves a balance between various objectives, such as noise suppression, speech quality preservation, and computational efficiency, by leveraging Pareto optimization in the deep learning model. The experimental results demonstrate the effectiveness of our approach in cleaning alaryngeal speech signals, making it a promising solution for various real-world applications.

## 1. Introduction

**Figure 1:**
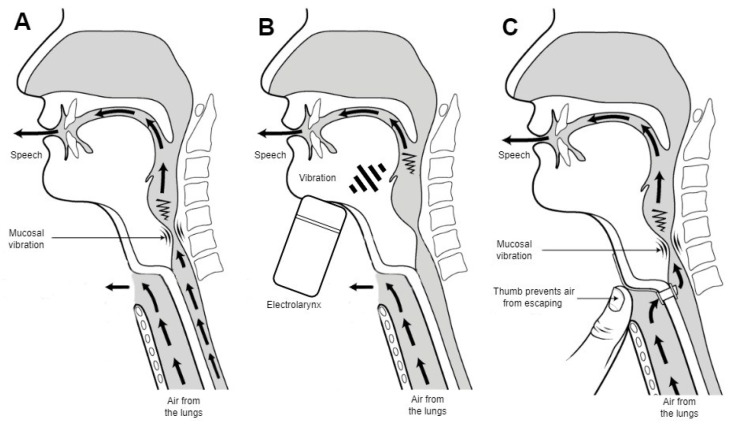
Types of speech production after total laryngectomy. (**A**) Esophageal speech: air is pulled in and released from the esophagus; (**B**) vibrations created by an electrolarynx; (**C**) the patient is occluding a tracheostoma to allow air to pass through the mouth. Adapted from Hurren 2015 [9].

Laryngeal cancer remains the most common malignant tumor in the upper respiratory tract [1]. Despite the decreasing incidence, approximately 60% of patients present with stage III or IV disease at the initial workup [2,3]. Surgery or surgery combined with chemoradiotherapy remains the preferred treatment method for laryngeal cancer, offering an optimal 5-year survival rate [4,5]. The surgical treatment options are laryngeal-preserving or radical surgery. Laryngeal-preserving surgery options can range from endolaryngeal cordectomy with a laser to partial removal of the larynx—partial laryngectomy. The complete removal of the larynx, also known as a total laryngectomy, is the radical option. The more advanced the disease, the more radical the treatment required to achieve remission. Advanced laryngeal cancer stages limit the treatment options that can be offered to patients. In most cases, the complete removal of the larynx—total laryngectomy—is advised. Such surgery leaves the patient without the larynx, the main part of the vocal apparatus, and their vocal function is significantly impaired. Long-term voice and speech function rehabilitation is required, often with unsatisfactory results. Total laryngectomy results in the complete and permanent separation of the upper and lower airways and requires the creation of a terminal tracheostoma to breathe. The complete removal of the larynx and lack of air movement through the mouth results in patients’ total loss of phonatory function [6]. After the removal of the larynx, the patient has to rely on alaryngeal speech to communicate. Alaryngeal speech can be achieved in three ways: esophageal speech, an electrolarynx, or a tracheoesophageal prosthesis (TEP). Esophageal speech and an electrolarynx benefit from low maintenance and do not require additional surgery. The TEP outperforms both methods by providing better perceptual (voice quality and intelligibility) and acoustic (maximum phonation time, fundamental frequency, and intensity) speech outcomes [7]. A TEP can be implanted through a tracheoesophageal fistula formed during laryngectomy or later [8]. It functions as a one-way valve that allows the air to move from the trachea to the esophagus but keeps the food and liquids from entering the lungs (see Figure 1).

**Figure 2:**
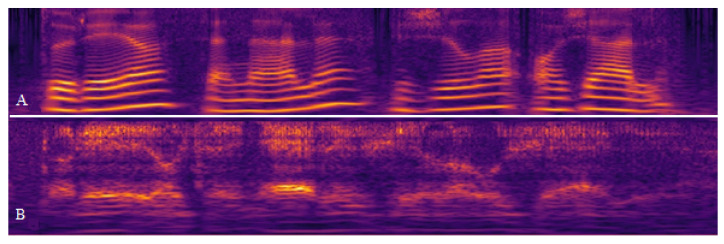
(**A**) Normal speech; (**B**) Speech after total laryngectomy with a tracheoesophageal prosthesis. Cochleagram deterioration and blurring between different words caused by aperiodicity, phonatory breaks, and additive noise.

The air moving through the TEP creates vibrations in the mucosa and generates speech [10]. The use of a pulmonary air supply to speak increases fluency and utterance lengths [11]. Despite its higher maintenance costs, the TEP is the preferred method for speech rehabilitation after total laryngectomy [12]. Alaryngeal (esophageal or TEP) speech is a patient’s only verbal communication option after a total laryngectomy. Although the patient retains the ability to speak, the body begins to adapt and substitutes vocal folds with structures (aryepiglottic/ventricular folds, pharyngeal mucosa) that were not naturally intended for voice production. The downside of this adaptation is that the speech generated in this manner features frequent unintended phonatory breaks, frequency shifts, unvoiced segments, and high irregularity, and might be aperiodic (see Figure 2) [13]. It becomes even more problematic when the patient has to use the phone or speak in a loud environment, which may lead to social isolation [14,15]. The inability to communicate is most prominent in the early postoperative period before any speech rehabilitation occurs when patients have to rely on written text to communicate with their physician and family.

It becomes even more problematic when the patient has to use the phone or speak in a loud environment, which may lead to social isolation [13,14]. The inability to communicate is most prominent in the early postoperative period before any speech rehabilitation occurs when patients have to rely on written text to communicate with their physician and family. Therefore, enhancing the signal quality of alaryngeal speech and improving a patient’s speaking ability represent fundamental scientific/technical and clinical issues.

This may involve techniques such as breath control [16], pitch and tone modifications [17,18], and articulation exercises [19], including spectral subtraction [20], Wiener filtering [21], and statistical prediction model-based [22] or machine learning-based approaches [23]. However, these traditional methods often suffer from drawbacks such as introducing artifacts, suppressing the desired speech components, or being computationally expensive. With the advent of deep learning, several new approaches have been proposed that leverage the power of neural networks to address the limitations of traditional methods. Among these, non-negative matrix factorization (NMF) [24] has gained significant attention for its ability to represent non-negative data such as audio spectrograms as a linear combination of basis functions [25].

In this paper, we propose a novel approach for cleaning impaired speech signals by combining Pareto-optimized deep learning with NMF. Our method aims to balance various objectives, such as noise suppression, speech quality preservation, and computational efficiency, by leveraging Pareto optimization in a deep learning model. By incorporating Pareto optimization, we ensure that the trade-offs between different objectives are optimally balanced, ultimately improving the performance of the noise-reduction process.

The proposed method consists of several steps. First, we calculate the spectrogram of a noisy voice clip and extract its frequency statistics. Based on the desired noise sensitivity, a threshold is calculated to distinguish between the signal and noise components in the spectrogram. Next, we compute the noise-to-signal mask and smooth it to avoid abrupt transitions in noise levels. The modified spectrogram is then obtained by applying the smoothed mask to the signal spectrogram. To further enhance the speech signal, we employ a Pareto-optimized NMF to decompose the modified spectrogram into basis functions and corresponding weights. These basis functions and weights are learned to best represent the clean speech signal while achieving a balance between various objectives. Finally, the clean speech spectrogram is reconstructed using the learned basis functions and weights, and a noise-reduced waveform is obtained by inverting the clean speech spectrogram.

The main contributions of this paper are as follows:

- We propose a novel method for cleaning impaired speech signals by combining Pareto-optimized deep learning with NMF, addressing the limitations of traditional speech enhancement techniques.
- We introduce a smoothing technique for the noise-to-signal mask to avoid abrupt transitions in noise levels, resulting in a more natural-sounding output signal.
- We demonstrate the effectiveness of our approach through a series of experiments, showing significant improvements in speech quality and intelligibility compared to traditional methods.

The remainder of this paper is organized as follows. Section 2 provides a review of the related works in the field of speech enhancement. In Section 3, we describe our proposed method. Section 4 presents the experimental setup and results, followed by a discussion of the findings. Finally, Section 5 concludes the paper and suggests future research directions.

## 2. Review of State-of-the-Art Works

This overview of related works aims to help the reader explore the various approaches to improving speech intelligibility and quality for individuals with speech disorders. Various techniques, such as clear speech variants, adaptive filter structures, deep learning models, and speech enhancement algorithms, have been investigated to address the challenges in speech enhancement. These studies demonstrate the potential of different methods, including instruction-based interventions, signal processing, and machine learning techniques, to enhance speech intelligibility and quality across various disorders and conditions. The findings may help the reader better understand the complex relationship between speech impairments and the effectiveness of different approaches in overcoming these challenges, ultimately improving communication for affected individuals.

### 2.1. Assessing Speech-Signal Impairments

Evaluating the quality and intelligibility of alaryngeal speech can be difficult for several reasons [26]. First, evaluating speech quality is inherently subjective, as different people may have different opinions on what constitutes good or clear speech. Evaluators may also have biases or preconceived notions about alaryngeal speech, which can affect their judgment [27]. Second, alaryngeal speech can be complex and variable, depending on the individual’s chosen method of alaryngeal speech and their level of proficiency [28], among other factors. Evaluators may need to consider multiple aspects of speech, such as pitch, tone, articulation, and prosody, which can make the evaluation more challenging [29]. Third, there is a limited amount of training data available for evaluating alaryngeal speech, as it is a relatively rare condition [30]. This can make it difficult to develop standardized evaluation methods and norms for different types of alaryngeal speech [31]. Finally, alaryngeal speech can vary widely between individuals, depending on factors such as age, gender, health status, and other individual characteristics [32]. This variability can make it difficult to develop standardized evaluation methods that are applicable to all individuals who have undergone a laryngectomy [33].

Numerous researchers have stressed the significance of selecting relevant characteristics for differentiating damaged speech [34,35,36]. Some researchers have investigated how speech difficulties caused by cerebral palsy and hearing impairment affect prosody, pronunciation, and voice quality. According to their findings, these factors are statistically significant for increasing the detection ability of impaired talks, with voice quality being the strongest discriminative feature for identifying speech intelligibility in damaged speech. Malini and Chandrakala [37] suggested a regularized self-representation-based compact supervector technique for assessing the intelligibility of damaged speech. On the UA-SPEECH database, their approach outperformed other methods such as hybrid GMM/SVM, supervector, x-vector, i-vector, and bag-of-models-based approaches. Albaqshi and Sagheer [38] emphasized the difficulties in dysarthric speech recognition owing to incomprehensible speech, irregular phoneme articulation, and data scarcity. Bessell et al. [39] found that a changed accent has a slower speech pace, greater consonant and vowel length, syllable-timed rhythm, and other characteristics. Moon et al. [40] sought to define the speech patterns of those suffering from hepatic encephalopathy as a possible diagnostic and monitoring tool. The subjects’ maintained and damaged speech patterns, on the other hand, did not follow patterns normally linked with organic brain problems, suggesting that left-handed preference may contribute to distinctions between singing and reading vs. recitation, repetition, and spontaneous speaking. This is also often the case after an ischemic stroke. De Cock et al. [41] investigated speech features, dysarthria type, and severity, showing that unilateral upper motor neuron dysarthria is the most common type, with the majority of subjects having mild dysarthria. Similarly, Rowe et al. [42] found that variable expressions of dysarthria may impact speech performance, whereas Stipancic and Tjaden [43] found the least detectable change in sentence intelligibility in speakers with multiple sclerosis and Parkinson’s disease. Rosdi et al. [44] presented fuzzy Petri nets to increase the classification accuracy of speech-intelligibility detection systems. Maskeliunas et al. suggested applying a convolutional network to help classify and asses impaired speech signals [45]. Kim et al. [46] used one- and two-dimensional convolutional neural networks to classify alaryngeal speech. Feng et al. [47] found that acoustic investigations can reveal that impaired speech has a substantially shorter voice start time for aspirated consonants, as well as a smaller vowel spacing. Vieira et al. [48] presented a non-intrusive voice-quality classifier based on the tree convolutional neural network for measuring user satisfaction with speech communication platforms. Poncelet et al. [49] suggested using an end-to-end spoken language understanding system that can be trained by the user through demonstrations and can translate impaired speech directly into semantics.

Numerous speech recognition-oriented techniques can also be used to help detect and asses speech impairment [50,51]. Gupta et al. [52] suggested a residual network-based approach for detecting dysarthria severity level based on short speech segments, whereas Latha et al. [53] employed deep learning and several acoustic cues to recognize dysarthric speech and generate discernible speech. Vishnika Veni and Chandrakala [54] researched the application of the deep neural network-hidden Markov model and lattice maximum mutual information technique for the successful identification of damaged speech. In [55], the authors suggested a histogram of states-based strategy for learning compact and discriminative embeddings for dysarthric voice detection using the deep neural network-hidden Markov model. Srinivasan et al. [56] proposed a multi-view representation-based disordered speech recognition system based on auditory image-based features and cepstral characteristics, showing improved performance in recognizing very low intelligibility words compared to conventional methods. Chandrakala et al. [57] presented a bag-of-models (BoM)-based approach that uses adjusted Gaussian mixture model (AGMM)-based embeddings for impaired speech-intelligibility evaluation. They tested the method on two datasets and discovered that it outperformed the supervector, hybrid GMM/SVM, i-vector, and x-vector-based techniques in terms of prediction error and reliability for intelligibility-level evaluation and score predictions. Fu et al. [58] created a Sch-net neural network built on a convolutional neural network for end-to-end schizophrenia speech identification using deep learning techniques, implying that it has the potential to help in the diagnosis of a particular language disability. Marini et al. [59] verified the efficacy of a speech analysis approach for dysarthria speakers by modifying the size and shift parameters of the spectral analysis window to increase ASR system performance.

### 2.2. Algorithms for Alaryngeal Speech Enhancement

The majority of voice restoration treatments result in hushed and monotonous speech. Aside from reduced intelligibility, this type of speech lacks expressiveness and naturalness due to (a) a lack of pitch, which results in whispered speech, and (b) artificial pitch production, which results in monotone speech. Algorithms for alaryngeal speech enhancement can be classified into two categories: classic digital signal processing (DSP) methods and methods based on artificial intelligence (AI) and machine learning (ML) [60].

The first category is the most popular as it includes filtering-based methods originally developed for noise reduction, as background noise can interfere with the clarity of alaryngeal speech [61]. DSP techniques, such as spectral subtraction, Wiener filtering, and adaptive filtering, can be used to reduce background noise and improve speech quality [62]. For example, Jaiswal et al. [63] suggested a concealed Wiener filter-based technique for voice augmentation to improve the common spectral subtraction algorithm. Pauline and Dhanalakshmi [64] presented an efficient adaptive filter structure for noise reduction in voice signals that utilized the least mean square (LMS) and normalized LMS algorithms. They evaluated the proposed filter model on both normal speech signals and speech signals from Parkinson’s disease patients. In terms of the SNR, MSE, and PSNR values, their filter model outperformed existing cascaded LMS filter models. Doi et al. studied how the LPC spectrum of alaryngeal speech could be used to determine the impulse response of the vocal tract. Modified harmonic amplitudes calculated using the transformation function were interpolated at the desired harmonics of the target pitch, and the transformation function was then computed using the line spectral frequencies rather than the harmonic amplitudes [65]. Pauline et al. [66] presented cascaded adaptive filter construction for speech-signal de-noising, where the best variable-stage cascaded adaptive filter model outperformed existing cascaded filter architectures, with an output SNR that was 10–15 dB higher. Panda et al. [67] suggested using spectral subtraction to improve alaryngeal speech, which was modified by Hamed et al. to include the power of noise [68]. Wei suggested using the Mel Frequency Scale as an alternative [69]. Another approach is pitch and formant manipulation, as alaryngeal speech can have a monotonous or robotic quality due to a lack of natural pitch and formant variation. DSP techniques such as pitch shifting and formant manipulation can also be used to add more natural-sounding variation to speech [70]. Giri and Rayavarapu [71] presented a combined approach for modifying the key frequency, intensity, and speech rate of dysarthric speech by utilizing time-domain pitch-synchronous overlap. They discovered that the improvement in intelligibility was significant in speakers with low initial intelligibility and modest in speakers with high intelligibility. Additionally, there are methods for articulation enhancement, as alaryngeal speech can also suffer from poor articulation, making it difficult to distinguish between different sounds. This can be combated by utilizing dynamic range compression, and equalization can be used to enhance the clarity and intelligibility of specific consonant sounds [72]. Finally, prosody modification is common, as it can help process the patterns of stress, intonation, and rhythm in speech. Alaryngeal speech can sometimes lack the natural prosody found in normal speech and prosody modification can be used to add more natural-sounding patterns of stress, intonation, and rhythm to speech [73].

The second category includes AI and ML methods that can be used for alaryngeal speech enhancement. Currently, the most popular methods are deep learning models [74], such as convolutional neural networks (CNNs), recurrent neural networks (RNNs), and generative adversarial networks (GANs), which can be trained on large datasets of alaryngeal speech to learn patterns and relationships between speech features and speech quality. These models can be used to perform classic DSP tasks such as noise reduction, pitch and formant manipulation, and prosody modification [75]. Saleem et al. [76] suggested a computationally efficient deep learning model for improving noisy voice. For magnitude estimation, their model used a U-shaped fuzzy long short-term memory, which outperformed other deep learning models and significantly enhanced speech intelligibility and quality. In contrast to traditional GAN-based approaches [77], Santjago et al. [78] suggested using a speaker-dependent GAN to enhance generated speech. Others have proposed that an adversarial acoustic regression loss should be added to encourage better extraction of features at the discriminator and employ a two-step adversarial training schedule that serves as a warm-up and fine-tune sequence. Both the objective and subjective assessments indicated that these two enhancements improved speech reconstruction by better matching the original speaker’s identity and naturalness [79]. Amarjuf combined the predicted phase with deep learning approaches to increase the overall quality [80]. Reinforcement learning can be applied to train speech enhancement systems that adapt to changing environments or input signals, as well as optimize speech enhancement systems based on a reward signal that reflects the quality of the enhanced speech [81]. Gaussian mixture models are also common, as they can work as a type of generative model that can be used to model the statistical distribution of speech features such as the spectral envelope or the fundamental frequency [82]. GMMs can also be used to separate speech from noise or modify the pitch and formant of speech. Ming et al. [83] suggested a hybrid technique that includes non-negative matrix factorization with GMM. According to Xui, the Gaussian mixture model approach is also beneficial for detecting vocal nodules and laryngitis [84]. Support vector machines can be used to classify impaired speech signals into different categories such as normal speech or alaryngeal speech produced using different methods [85]. SVMs can also be used for noise reduction and speech enhancement [86]. Hidden Markov models can also be used as a generative model that models the statistical distribution of speech features over time, often to classify speech signals into different categories or generate new speech signals based on the statistical distribution of the input speech [87].

## 3. Materials and Methods

### 3.1. Dataset

Thirty native Lithuanian-speaking male patients surgically treated for histologically confirmed laryngeal cancer at the Lithuanian University of Health Sciences Department of Otorhinolaryngology provided speech samples for this study. The patients in this group had undergone a total laryngectomy with secondary TEP implantation [88,89]. These individuals were chosen because they had no larynx or vocal folds and relied solely on alaryngeal speech to communicate. The complete removal of the larynx and speech production using a TEP often result in distinct speech abnormalities with a fairly uniform functional speech handicap compared to neurodegenerative disorders, where speech patterns are more diverse and less distinct. The average age of the patients was 63.1 years (standard deviation = 28.8). The patients were free of common colds, upper respiratory infections, or other conditions that may have affected speech quality at the time of recording. Only male participants’ speech samples were collected since advanced laryngeal cancer is less common in women, and recruiting an adequate number of female participants was not feasible. Endoscopic evaluation of the neopharynx, TEP canal, and trachea was performed prior to recording. Faulty or leaking prostheses were replaced prior to recording. This examination was carried out as part of standard clinical practice and contributed to the speech sample database exclusively containing speech samples from patients in remission. For at least six months following surgery, speech recordings were acquired. This ensured enough time for healing, speech adaptation, and rehabilitation [90].

Alaryngeal speech samples were recorded in a T-series quiet room (T-room, CA Tegner AB, Bromma, Sweden) using a D60S Dynamic Vocal microphone (AKG Acoustics, Vienna, Austria) placed 10.0 cm from the lips at a comfortable (about 90°) microphone-to-mouth angle. Two different speaking assignments were completed. The patient began by reading a phonetically balanced Lithuanian line: “Turėjo senelė žilą oželį” (Old grandma had a billy goat). The relative frequencies of the phonemes in the phrase were made as close as possible to the distribution of speech sounds in Lithuanian. The patient then counted from one to ten at a rate appropriate for their respiratory function. All speech activities were performed at a comfortable volume level and at the patient’s own tempo. Speech was recorded at 44,100 samples per second and saved as uncompressed 16-bit waveform audio format files. Using Praat version 6.0.53, the recordings were manually prepared and contained no more than 300 ms of an unvoiced fragment at the beginning and conclusion of the recordings. To ensure the security of participants’ personal data, serial numbers were assigned to the speech recordings.

### 3.2. Alaryngeal Speech Assessment

Several approaches were used to measure objective alaryngeal speech:

1. The artificial intelligence-based automated classifier for substitution voicing ResNet 118 was used to assign speech samples to the following classes: normal speech—Probability 0; speech with a single vocal fold—Probability 1; and alaryngeal speech with TEP—Probability 2 [91].
2. The acoustic parameter of alaryngeal speech (average voicing evidence (AVE), available in the AMPEX software [92]) was utilized to compare the alaryngeal speech samples before and after optimization using Pareto-optimized NMF software. The AVE parameter describes the average voicing evidence and the degree of regularity/periodicity in the voiced frames. Since the actual background frames are usually unvoiced, the analysis is performed on all frames, not just speech frames. This approach is more robust against possible errors of the speech/background classification, which is purely energy-based. In contrast, the voicing evidence is derived from analyzing all the sub-band signals created by the auditory model.
3. The AI-based acoustic substitution voicing index (ASVI) parameter [93] was employed to quantitatively evaluate the alaryngeal speech samples before and after optimization using Pareto-optimized NMF software. This parameter includes the constant combined with statistically significant parameters from ResNet 118 (Probability 0, Probability 1, and Probability 2) combined with the AVE and mean fundamental frequency. The possible ASVI values ranged from 0 to 30, with better speech quality indicated by higher scores.

### 3.3. Methodology

Our approach used Pareto-optimized deep learning to evaluate the possibility of cleaning the impaired speech. The approach started by calculating the spectrogram over the entire noisy voice clip, based on which the frequency statistics were calculated. Once the statistics were calculated, a threshold based on the desired noise sensitivity was then calculated. Afterward, a signal spectrogram was calculated based on the same input noisy voice clip, which, in combination with the calculated threshold, was then used to determine the noise-to-signal mask. The mask was then smoothed by applying a filter in both frequency and time to avoid sudden jumps in noise levels. Finally, the smoothed mask was then applied to the spectrogram of the signal and inverted creating a noise-reduced waveform.

#### 3.3.1. Non-Negative Matrix Factorization (NMF)

Given a non-negative matrix V $\in R_{\geq 0}^{m\times n}$, non-negative matrix factorization (NMF) aims to find two non-negative matrices W $\in R_{\geq 0}^{m\times k}$ and H $\in R_{\geq 0}^{k\times n}$ such that their product approximates the original matrix V:

(1) V≈WH

The objective is to minimize the distance between V and WH, typically measured by the Frobenius norm or another divergence measure:

(2) $$\min_{W\geq 0,H\geq 0}∥V---WH∥$$

where ∥·∥ denotes the Frobenius norm or another divergence measure, and *k* is the desired dimensionality of the factorization (typically, k≪min(m,n)).

#### 3.3.2. Pareto-Optimized Non-Negative Matrix Factorization (PONMF)

We define Pareto-optimized NMF as the problem of approximating a non-negative matrix V with the product of two non-negative matrices W and H, considering multiple objectives $f_{1},f_{2},\ldots ,f_{p}$. The Pareto-optimized NMF formulation seeks a solution that balances the trade-offs among these objectives, achieving a Pareto optimal solution where no objective can be improved without worsening at least one other objective.

Given a non-negative matrix V $\in R_{\geq 0}^{m\times n}$, Pareto-optimized non-negative matrix factorization (NMF) aims to find two non-negative matrices W $\in R_{\geq 0}^{m\times k}$ and H $\in R_{\geq 0}^{k\times n}$ such that their product approximates the original matrix V:

(3) V≈WH

The objective is to find a Pareto optimal solution, considering multiple objectives $f_{1},f_{2},\ldots ,f_{p}$. A Pareto optimal solution is one where it is not possible to improve any objective without worsening at least one other objective. The Pareto-optimized NMF can be formulated as:

(4) $$\min_{W\geq 0,H\geq 0}\left(f_{1}\left(V,W,H\right),f_{2}\left(V,W,H\right),\ldots ,f_{p}\left(V,W,H\right)\right)$$

subject to Pareto optimality. Here, $f_{i}\left(V,W,H\right)$ represents the *i*-th objective such as minimizing the reconstruction error, promoting sparsity, or reducing computational complexity. The goal is to find a solution that balances the trade-offs among these objectives.

The first step is to calculate the spectrogram over the entire noisy voice clip to obtain a representation of the frequency spectrum of a signal over time. The noisy voice clip is windowed and its Fourier transform is calculated to obtain a spectrogram.

Once the spectrogram is calculated, frequency statistics are calculated to obtain a better understanding of the frequency distribution of the signal. This is achieved by calculating the mean and standard deviation of the magnitude of each frequency bin over time.

**Figure 3:**
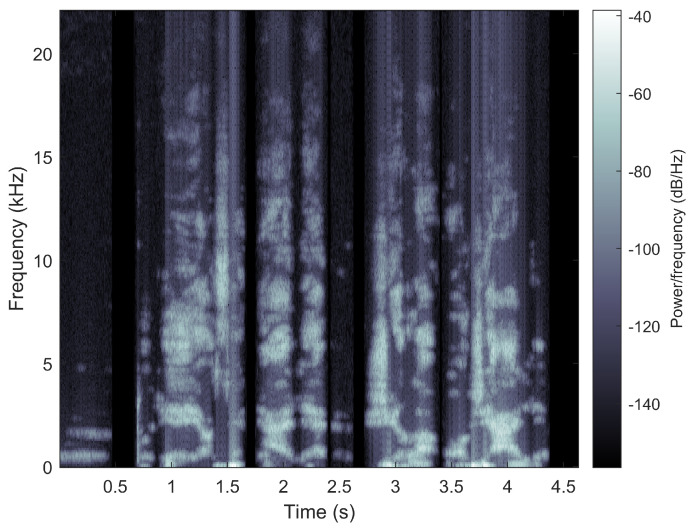
An example of a voice spectrogram.

Based on the desired noise sensitivity, a threshold is calculated to distinguish between the signal and noise in the spectrogram. A signal spectrogram (see an example in Figure 3) is then calculated based on the same input noisy voice clip. This is achieved by windowing the noisy voice clip and taking its Fourier transform over time. The threshold calculated earlier is used to determine the noise-to-signal mask. The mask is a binary value for each frequency bin and time frame of the spectrogram, where 1 indicates the signal and 0 indicates noise. To avoid sudden jumps in noise levels, the mask is smoothed by applying a filter in both the frequency and time domains, making the noise-to-signal mask more continuous and less abrupt. Next, the smoothed mask is applied to the spectrogram of the signal, and the signal is inverted to create a noise-reduced waveform. This is achieved by multiplying the spectrogram of the signal with the smoothed mask and then taking the inverse Fourier transform over time to obtain the noise-reduced waveform. Then, a Pareto-optimized non-negative matrix factorization (NMF)-based method is applied to decompose the spectrogram into a set of basis functions and their corresponding weights. NMF-based methods for speech enhancement involve learning the basis functions and Pareto-optimized weights that best represent the clean speech signal and then using these to reconstruct the clean speech from a noisy input signal (Algorithm 1).
**Algorithm 1** Pareto-Optimized Deep Learning for Impaired Speech Cleaning**Require:** Noisy voice clip *V***Ensure:** 
Noise-reduced waveform *W*  1:Calculate spectrogram *S* of noisy voice clip *V*  2:Compute frequency statistics *F* from spectrogram *S*  3:Calculate threshold *T* based on the desired noise sensitivity using frequency statistics *F*  4:Determine signal spectrogram $S_{signal}$ using noisy voice clip *V*  5:Compute noise-to-signal mask *M* using threshold *T* and signal spectrogram $S_{signal}$  6:Smooth mask *M* by applying a filter in both the frequency and time domains to obtain smoothed mask $M_{smooth}$  7:Apply smoothed mask $M_{smooth}$ to the spectrogram of signal $S_{signal}$ to obtain modified spectrogram $S_{mod}$  8:Invert modified spectrogram $S_{mod}$ to create noise-reduced waveform *W*   9:**return** *W*

#### 3.3.3. Speech-Signal Cleaning

The updated approach to cleaning impaired speech using Pareto-optimized deep learning and non-negative matrix factorization (NMF) involves the following steps:

1. Calculate the spectrogram of the entire noisy voice clip. This is achieved by windowing the noisy voice clip and taking its Fourier transform over time to obtain a spectrogram, which is a representation of the frequency spectrum of a signal over time.
2. Compute the frequency statistics from the spectrogram. This is achieved by calculating the mean and standard deviation of the magnitude of each frequency bin over time. These statistics help in understanding the distribution and characteristics of the noise present in the voice clip.
3. Calculate a threshold based on the desired noise sensitivity. This threshold helps differentiate between the noise and signal components in the spectrogram.
4. Determine the signal spectrogram using the same input noisy voice clip. This is achieved by windowing the noisy voice clip and taking its Fourier transform over time.
5. Compute the noise-to-signal mask using the calculated threshold. The mask is a binary value for each frequency bin and time frame of the spectrogram, where 1 indicates the signal and 0 indicates noise.
6. Smooth the noise-to-signal mask by applying a filter in both the frequency and time domains. This helps avoid sudden jumps in noise levels and produces a more continuous and less abrupt mask.
7. Apply the smoothed mask to the spectrogram of the signal. This step effectively suppresses the noise components in the spectrogram while retaining the desired signal.
8. Decompose the modified spectrogram using Pareto-optimized non-negative matrix factorization (NMF). NMF-based methods for speech enhancement involve learning the basis functions and Pareto-optimized weights that best represent the clean speech signal.
9. Reconstruct the clean speech from the noisy input signal using the learned basis functions and Pareto-optimized weights.
10. Invert the reconstructed spectrogram to create a noise-reduced waveform. This final output is a cleaned version of the original impaired speech, with the noise components significantly reduced or removed.

#### 3.3.4. Pareto-Optimized Deep Learning with NMF for Impaired Speech Cleaning

Using Pareto optimization in the deep learning model and incorporating NMF-based methods can ensure that the trade-offs between different objectives (e.g., noise suppression, speech quality, and computational efficiency) are balanced in the best possible way, ultimately improving the performance of the noise-reduction process (Algorithm 2).
**Algorithm 2** Pareto-Optimized Deep Learning with NMF for Impaired Speech Cleaning**Require:** 
Noisy voice clip *V***Ensure:** 
Noise-reduced waveform *W*  1:Calculate spectrogram *S* of noisy voice clip *V*  2:Compute frequency statistics *F* from spectrogram *S*  3:Calculate threshold *T* based on the desired noise sensitivity using frequency statistics *F*  4:Determine signal spectrogram $S_{signal}$ using noisy voice clip *V*  5:Compute noise-to-signal mask *M* using threshold *T* and signal spectrogram $S_{signal}$  6:Smooth mask *M* by applying a filter in both the frequency and time domains to obtain smoothed mask $M_{smooth}$  7:Apply smoothed mask $M_{smooth}$ to the spectrogram of signal $S_{signal}$ to obtain modified spectrogram $S_{mod}$  8:Decompose modified spectrogram $S_{mod}$ using Pareto-optimized non-negative matrix factorization (NMF) to obtain basis functions *B* and optimized weights $W_{opt}$  9:Reconstruct clean speech spectrogram $S_{clean}$ using basis functions *B* and optimized weights $W_{opt}$10:Invert clean speech spectrogram $S_{clean}$ to create noise-reduced waveform *W*

## 4. Results

**Figure 4:**
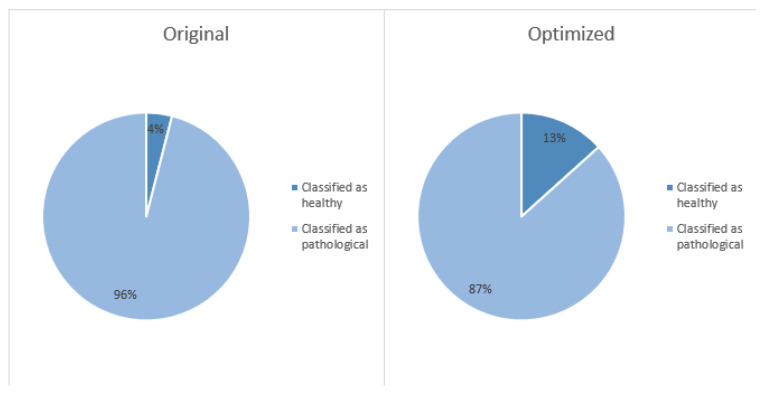
A pie chart illustrating the proportion of speech recordings classified as normal/pathological before and after speech-signal optimization.

A statistically significant improvement in alaryngeal speech quality was observed because after applying Pareto-optimized NMF, the alaryngeal speech samples were reclassified into the lower speech disability category (see Table 1 and Figure 4).

To further test for improvements in the optimized speech samples, the Chi-squared test [94] was utilized to test if the proportion of speech recordings considered improved was large enough to be statistically significant. Only 4 out of 75 original speech recordings were classified as healthy, whereas 10 out of 75 were classified as healthy speech after optimization. This resulted in a statistically significant difference between the proportions (*p* = 0.043). These findings can be observed in Table 2.

**Figure 5:**
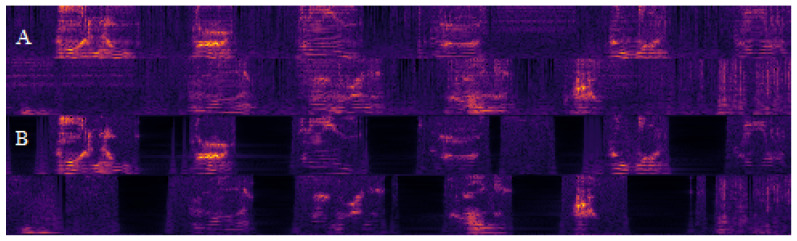
Cochleograms depicting a patient counting to ten with a tracheoesophageal prosthesis before (**A**) and after (**B**) speech-signal optimization. Less additive noise between separate numbers can be observed in the optimized cochleagram.

An example of the result of the alaryngeal speech-signal optimization is presented in Figure 5.

Table 3 presents the results of statistical tests (Levene’s test and t-test) performed on several groups of data (Probability 0, Probability 1, Probability 2, AVE, and ASVI).

Levene’s test for equality of variances checks whether the variances are equal across the groups. The null hypothesis is that the variances are equal. If the significance (sig.) is less than the threshold level (commonly 0.05), the null hypothesis is rejected, indicating that the variances are not equal. The choice between “equal variances assumed” and “equal variances not assumed” is determined by the results of Levene’s test. If the variances are found to be equal (sig. > 0.05 in Levene’s test), then we should refer to the t-test row for “equal variances assumed”. If the variances are not equal (sig. < 0.05 in Levene’s test), we should refer to the row “equal variances not assumed”. The significance for each group of data was as follows:

- Probability 0: sig. = 0.000, indicating that the variances were not equal across groups.
- Probability 1: sig. = 0.454, indicating that the variances were equal.
- Probability 2: sig. = 0.008, indicating that the variances were not equal.
- AVE: sig. = 0.340, indicating equal variances across groups.
- ASVI: sig. = 0.166, indicating equal variances across groups.

The *t*-test for equality of means checks whether the means of two groups are statistically significantly different. The null hypothesis is that the means are equal. If the significance (two-tailed) is less than the threshold level (commonly 0.05), the null hypothesis is rejected. The significance value for each group of data was as follows:

- Probability 0: sig. = 0.036 (for equal variances assumed) and 0.037 (for equal variances not assumed), indicating that the means of the two groups were significantly different.
- Probability 1: sig. = 0.890 (both cases), indicating that the means were not significantly different.
- Probability 2: sig. = 0.163 (both cases), indicating that the means are not significantly different.
- AVE: sig. = 0.750 (both cases), indicating that the means are not significantly different.
- ASVI: sig. = 0.133 (for equal variances assumed) and 0.134 (for equal variances not assumed), indicating that the means are not significantly different.

The mean difference, standard error difference, and 95% confidence interval of the difference provide further details on how the means of the two groups differed and the uncertainty surrounding that difference.

To summarize, as shown in Table 1, the mean AVE of the alaryngeal speech samples decreased from 81 to 80% after optimization. The AVE proportion remained statistically significant and unchanged in the samples before and after Pareto–NMF optimization. This is understandable and expected because the Pareto-optimized NMF approach removed background noise without artificially improving the quality of the alaryngeal speech recordings by filling in the unvoiced speech segments (pauses, intended phonatory breaks, etc.).

Lastly, the speech samples were evaluated using the ASVI, which represents the scale of the objective improvement of the alaryngeal speech signals when comparing the original and Pareto-optimized NMF alaryngeal speech recordings. Although the ASVI was higher in the group after optimization, the difference was not statistically significant. A description of the aforementioned evaluation can be found in Table 1.

## 5. Discussion

Speech is the complex result of several systems in the body working together. First, the respiratory tract must move air through the larynx and mouth. The vocal folds need to function correctly to produce voice. Speech is produced only when the articulation occurs in the pharynx and mouth and is then processed by the speaker’s neural feedback loop, which helps correct the pitch and loudness. Finally, speech is used to communicate, so it has to be pleasant or, at the very least, intelligible to the listener [95]. Disturbances in any of these steps cause various levels of speech impairment.

Total laryngectomy patients often undergo speech rehabilitation programs to learn alternative methods of speech production such as esophageal speech, an electrolarynx, or tracheoesophageal speech with a voice prosthesis. These techniques can generate additional noise during speech production, thereby affecting speech quality and intelligibility. Implementing noise-reduction strategies can help mitigate this issue by improving the overall clarity and naturalness of the patient’s speech [7]. However, a speech handicap becomes more problematic when the patient has to use the phone or speak in a loud environment, which may lead to social isolation [14,15].

The suggested Pareto–NMF optimization approach helps mitigate the additive and background noise problem that is common in alaryngeal speakers. The Pareto–NMF optimization removes additive and background noise without impacting the AVE. Although minuscule for a regular speaker, this improvement benefits the TEP speaker significantly. Firstly, total laryngectomy patients often face challenges in making their speech intelligible, especially in noisy environments. Excessive background noise can mask their already limited vocal output, making it difficult for listeners to understand them. By reducing the additional noise present in the environment, speech clarity and intelligibility can be improved, allowing patients to communicate more effectively. Secondly, speaking on the phone can be particularly challenging for individuals after laryngectomy [96]. Background noise, distortions, and limited vocal output can make it difficult for the listener to comprehend the speech. Unwanted noise can be minimized by implementing noise-reduction techniques, enabling clearer and more understandable phone conversations for laryngectomy patients.

Minimal Pareto–NMF optimization impact on speech benefits perfect TEP speakers more, as the spoken segments are largely unaltered and rely solely on the speaker’s ability to speak clearly. Patients who have trouble articulating with a TEP could potentially benefit more from Pareto–NMF optimization combined with a speech enhancement model that addresses unvoiced segments, aperiodicity, and phonatory breaks that are more frequent in less experienced alaryngeal speakers.

A typical laryngeal cancer patient eligible for total laryngectomy and TEP rehabilitation is between 50 and 70 years of age and rarely has significant comorbidities [97,98]. After successful treatment, it is reasonable to expect at least a 40% 5-year survival rate. The combination of these conditions leads to a rather specific problem—a large group of patients that are functionally able to return to completely normal life or even the workforce but are held back by their speech disability. Alaryngeal speech enhancement techniques can help mitigate this problem and to allow complete rehabilitation and reintegration for patients after total laryngectomy.

## 6. Conclusions

Speech after surgical treatment for laryngeal cancer tends to suffer from aperiodicity, phonatory breaks, and additive noise [90]. These findings become more common as more laryngeal structures are removed. However, the adaptive capabilities of patients can result in vastly different acoustical outcomes despite undergoing identical surgery. This is reflected in the relatively high standard deviation observed when evaluating the ASVI of original and optimized speech samples. With this in mind, studies on acoustic speech after laryngeal oncosurgery should be carried out with a greater number of recordings.

## Figures and Tables

**Table 1:** Evaluation results of original and optimized speech samples. Prob0—probability of healthy speech; Prob1—probability of speech with a single vocal fold; Prob2—probability of tracheoesophageal speech; AVE—average voicing evidence; ASVI—acoustic substitution voicing index; NMF—non-negative matrix factorization.

	Group	N	Mean	Std. Deviation	*p*
Probability 0	Original	75	4.09	19.51	0.001
	Pareto-optimized NMF	75	13.51	33.3	0.001
Probability 1	Original	75	56.18	48.66	0.454
	Pareto-optimized NMF	75	57.28	47.83	0.454
Probability 2	Original	75	39.73	47.9	0.08
	Pareto-optimized NMF	75	29.21	43.89	0.08
AVE	Original	75	0.81	0.11	0.34
	Pareto-optimized NMF	75	0.8	0.1	0.34
ASVI	Original	75	8.8	4.94	0.166
	Pareto-optimized NMF	75	10.17	6.09	0.166

**Table 2:** Comparison of original and optimized speech recordings. NMF—non-negative matrix factorization.

Group	Method	N	*p*	$\chi^{2}$
Healthy speech	Original	4	4.0	0.043
	Pareto-optimized NMF	10	13.33	
Speech after laryngeal oncosurgery	Original	72	96.0	4.097
	Pareto-optimized NMF	65	86.67	

**Table 3:** Results of statistical tests.

		Levene’s Test		*t*-Test for Equality of Means						
		F	Sig.	t	df	Sig. (2-Tailed)	Mean Difference	Std. Error Difference	95% Conf. Int.	
									Lower	Upper
Probability 0	Equal variances assumed	18.313	0.000	−2.113	148	0.036	−9.41893	4.45670	−18.22592	−0.61195
	Equal variances not assumed			−2.113	119.448	0.037	−9.41893	4.45670	−18.24330	−0.59457
Probability 1	Equal variances assumed	0.563	0.454	−0.139	148	0.890	−1.09627	7.87862	−16.66538	14.47284
	Equal variances not assumed			−0.139	147.956	0.890	−1.09627	7.87862	−16.66542	14.47288
Probability 2	Equal variances assumed	7.317	0.008	1.402	148	0.163	10.51547	7.50161	−4.30864	25.33957
	Equal variances not assumed			1.402	146.885	0.163	10.51547	7.50161	−4.30957	25.34050
AVE	Equal variances assumed	0.918	0.340	0.319	148	0.750	0.005560	0.017451	−0.028926	0.040046
	Equal variances not assumed			0.319	147.237	0.750	0.005560	0.017451	−0.028927	0.040047
ASVI	Equal variances assumed	1.941	0.166	−1.509	148	0.133	−1.36607	0.90525	−3.15495	0.42281
	Equal variances not assumed			−1.509	141.961	0.134	−1.36607	0.90525	−3.15558	0.42343

## Data Availability

The data presented in this study are available in this article.

## Abbreviations

The following abbreviations are used in this manuscript:

NMF	non-negative matrix factorization
UA-SPEECH	sound dataset
GMM	Gaussian mixture model
SVM	support vector machine
AGMM	adjusted Gaussian mixture model
DSP	digital signal processing
AI	Artificial Intelligence
LMS	least mean square
SNR	signal-to-noise ratio
MSE	mean square error
PSNR	peak signal-to-noise ratio
LPC	linear predictive coding
CNN	convolutional neural network
RNN	recurrent neural network
GAN	generative adversarial network
TEP	tracheoesophageal prosthesis
Prob0	probability of healthy speech
Prob1	probability of speech with a single vocal fold
Prob2	probability of tracheoesophageal speech
AVE	average voicing evidence
ASVI	acoustic substitution voicing index
